# Patients' views and experiences about unused medications and potential interventions to reduce medication waste

**DOI:** 10.1016/j.rcsop.2026.100859

**Published:** 2026-09-30

**Authors:** Dawit T. Gebregeorgise, Teferi G. Fenta, Bart J.F. van den Bemt, Charlotte L. Bekker

**Affiliations:** a Social and Administrative Pharmacy, School of Pharmacy, College of Health Sciences, Addis Ababa University, Addis Ababa, Ethiopia; b Department of Pharmacy, Sint Maartenskliniek, Nijmegen, the Netherlands; c Department of Pharmacy, pharmacology and toxicology, Radboud University Medical Center, P.O. Box 9101, 6500 HB Nijmegen, the Netherlands

**Keywords:** Patients' perspectives, Unused medications, Potential interventions, Medication waste, Ethiopia, Primary healthcare

## Abstract

**Background:**

Patients frequently end up with unused medications due to non-adherence, excessive supply, treatment changes, or early discontinuation. However, patients' views on unused medications and potential waste minimization interventions remain underexplored in low- and middle-income countries. This study aimed to explore patients' views and experiences regarding unused medications and potential solutions for reducing the amount of unused medications in homes.

**Methods:**

Semi-structured interviews (*n* = 26) were conducted with adult patients who visited a health facility and were using prescription medication. Participants were asked about their perceptions of unused medications, their handling and disposal of unused medications, and potential interventions to reduce medication waste. Interviews were transcribed verbatim and analyzed using inductive thematic analysis.

**Results:**

Patients' views of unused medications were both positive and negative. Positive views included potential use in future disease management, while negative views included risks of accidental poisoning, misuse, environmental pollution, and financial loss. Key factors contributing to unused medications included patient non-adherence, health system gaps, and poor patient-healthcare provider relationships. Notably, most patients disposed of unused medications in open fields. To mitigate this waste, proposed solutions focused on patient education and counselling regarding responsible use and safe disposal, equipping public health facilities with the necessary diagnostic equipment, and strengthening health care providers' technical knowledge and skills.

**Conclusion:**

Patients have mixed views regarding unused medications kept at home, and improper disposal practices remain widespread. To minimize medication waste and its associated risks, interventions should focus on improving patients' medication use behaviour. Specifically, there is a critical need for patient education and counselling on the risks and benefits of unused medications, along with strategies to strengthen patients' relationships with healthcare providers.

## 1 Introduction

Unused medications are defined as expired, damaged or leftover medications that are kept but not needed for future use.^1^ A significant amount of medications remain unused in patients' homes.^2^, ^3^, ^4^, ^5^ Non-adherence,^6^ pharmacy oversupply, changes in therapy, and early treatment discontinuation are among the most commonly reported reasons for unused medication accumulation.^7^

This poses safety risks, particularly when unused medications are stored or disposed of unsafely, shared, or misused. Furthermore, unused medications can have negative economic consequences, including significant financial losses, especially for expensive medications such as oral anticancer drugs.^8^, ^9^, ^10^ Additionally, improper disposal of these medications can lead to environmental pollution.^11^, ^12^

Although low- and middle-income countries consume relatively few medications,^13^, ^14^, ^15^ research shows that these countries face disproportionate health,^16^ economic, and environmental^16^, ^17^ risks associated with unused medications. Several studies report the prevalence of unused medications per home, ranging from 38% to 99.6%, with potential and real wastage of 53.8% and 38.8%, respectively.^18^ Low- and middle-income countries are challenged by an unsecured medicine supply chain; weak regulation and enforcement^16^, ^17^, ^19^; poor infrastructure^16^ for safe disposal and waste management; and low awareness of safe disposal methods.^20^ Measures are needed to minimize the sharing, misuse, or unsafe disposal of unused medications.^19^

A coordinated response from key stakeholders, including manufacturers, distributors, health authorities, prescribers, and dispensers, not only ensures a sustainable supply and responsible use of pharmaceuticals but also minimizes waste.^21^ For instance, pharmacists can play a crucial role in waste-reduction efforts by conducting medication reviews at the point of dispensing, optimizing their own stock management and raising patients' awareness about medication waste.^22^ However, pharmacist engagement in such prevention activities is limited by high workloads, and a lack of training and education.^23^

In Ethiopia, previous studies have quantitatively assessed patients' knowledge, attitudes, and disposal practices regarding unused medications,^5^, ^24^, ^25^, ^26^, ^27^, ^28^, ^29^ and, have explored how public health facilities manage medication waste.^27^ These studies showed that most patients were unaware of how to prevent the accumulation of unused medications or how to dispose of them safely,^24^, ^26^, ^30^ even when they were aware of the potential harms of inappropriate disposal practices.^24^ However, these studies did not qualitatively explore the subjective experiences and meanings that patients living in remote settings attach to these medications. Understanding these views is critical for gaining deeper insight into patient behaviour and effectively engaging them when designing and implementing patient-focused waste-minimizing measures. Therefore, this study aimed to explore patients' views and experiences regarding unused medications and potential solutions to prevent them from accumulating in patients' homes.

## 2 Methods

### 2.1 Study design and setting

A phenomenological qualitative study was employed, using semi-structured individual interviews. The Consolidated Criteria for Reporting Qualitative Research (COREQ) checklist was used to ensure explicit and comprehensive reporting.^31^

The study was conducted in North Shoa Zone, Amhara Regional State, which is the second largest regional state in Ethiopia.^32^ The zone has one referral hospital, two general hospitals, seven primary hospitals, and 91 health centres and 37 health posts. Water and electricity supplies are available in only 70% and 86% of health centers, respectively, and in only 10% and 9% of health posts. Infectious diseases, along with maternal and neonatal health issues and malnutrition, account for the highest share of morbidity and mortality.^33^ The zone also has no drug take-back program.

On average, 2,142,495 outpatients visit public health facilities annually. Of these, 86.9% receive care in the primary health care unit (PHCU),^33^ the first tier of the healthcare system, comprising primary hospitals, health centres and health posts. PHCUs operate at the woreda (district) and kebele (lowest administrative) levels to provide primary health care services to communities.^34^ Three health centres with the highest patient loads, which form part of an ongoing multi-center PhD project,^5^ were included, and patients were recruited from these centres.

### 2.2 Participant selection

Patients were eligible for inclusion if they: (1) were aged 18 years and older, (2) had received medication at one of the selected health facilities and used that medication at home during the three months before data collection, (3) were willing to be interviewed, and (4) spoke the local language (i.e., Amharic). A maximum variation sampling technique was applied to capture a broad range of perspectives. Variation in sex, age, educational level, and the presence of chronic and acute conditions was considered when recruiting patients to ensure a diverse population. Patients who had been diagnosed with HIV/AIDS (because of the risk of stigmatisation) or who were seriously ill at the time of recruitment were excluded. Eligible patients were approached by the healthcare professionals (i.e., treating physicians) working at the selected data collection sites, and interested patients were then contacted by the principal investigator (PI), DTG, for a face-to-face interview. Before initiating the interview, participants were asked to provide verbal consent.

### 2.3 Data collection

Based on previous literature and the investigators' experience, a semi-structured interview guide was developed and pilot tested with five patients with chronic disease to check whether the questions were clear and comprehensive. The interview guide covered three domains: patient perceptions of unused medications, the handling and disposal of unused medications, and potential interventions to reduce medication waste. Items that were not clear to the interviewees were subsequently modified. All interviews were conducted between March and April 2023 by the PI (DTG), a PhD candidate with prior training and experience interviewing patients. DTG, a male pharmacist, had no prior relationship with study participants. During the interviews, one participant requested that her husband be present, and another was interviewed in the presence of her child. In all other interviews, no one apart from the interviewer and interviewee was present. Consenting patients' background information, such as age, education, and presence of unused medication(s), was recorded. In addition, the interviews explored their views about unused medications; the risks, problems and benefits of keeping unused medications; the perceived causes of and solutions for medication waste; and the roles that can be played by key actors such as patients, prescribers, dispensers, and health authorities in the prescribing, dispensing and use of medication to minimize medication waste. All interviews were conducted in the local language at the three health centres and were audio-recorded.

### 2.4 Data analysis

Inductive thematic analysis was used following Braun and Clarke's framework.^35^ First, all interview recordings were transcribed into Amharic, translated into English by an English-proficient pharmacist (interpreter), and subsequently verified against the original audio by the first author. All transcripts were read, and the audio recordings were listened to repeatedly to gain an overall understanding of the data. Following this, key aspects in the data items across the data set were labelled with open codes in ATLAS.ti 24.0.0 by the PI (DTG) and reviewed by a second researcher (CB). These codes were categorised, and the PI identified potential themes; these were discussed iteratively with the research team until consensus was reached. Finally, an analytical narrative was compiled, integrating illustrative quotes to represent the findings clearly.

### 2.5 Ethical clearance

The Ethics Committee of the School of Pharmacy, Addis Ababa University [ERB/SOP/466/15/2023] granted ethical approval for this qualitative study. Permission to conduct the study was also obtained from the North Shoa Zonal Health Bureau, the selected Woreda Health Bureaus, and the participating health centres, and verbal consent was obtained from all patients.

## 3 Results

In total, 26 patients were approached and all agreed to be interviewed. Of these, one discontinued the interview after it had started because they felt sick and were in pain. Most interviewees were female (73%) and had no formal education (58%) and their ages ranged from 20 to 58 years (median 43.5 years) Furthermore, just over half of the sample (54%) reported having had unused medications in the past (Table 1).

**Table 1:** Background characteristics of respondents.

Participant	Sex	Age	Education	Presence of unused medication
Participant 1	Female	20	Secondary School	Yes
Participant 2	Female	49	Secondary School	Yes
Participant 3	Female	52	No education	Yes
Participant 4	Female	45	Primary School	No
Participant 5	Female	26	Secondary School	No
Participant 6	Female	42	Secondary School	Yes
Participant 7	Female	48	No education	No
Participant 8	Female	40	No education	No
Participant 9	Female	42	No education	Yes
Participant 10	Female	38	No education	No
Participant 11	Female	38	No education	No
Participant 12	Female	45	No education	Yes
Participant 13	Female	25	Secondary School	Yes
Participant 14	Female	47	No education	Yes
Participant 15	Male	49	No education	No
Participant 16	Male	38	Primary School	No
Participant 17	Male	58	No education	No
Participant 18	Male	45	No education	No
Participant 19	Male	32	Secondary School	No
Participant 20	Female	48	No education	No
Participant 21	Male	20	Primary School	Yes
Participant 22	Female	26	Degree	Yes
Participant 23	Male	28	Degree	Yes
Participant 24	Female	52	No education	Yes
Participant 25	Female	57	No education	Yes
Participant 26	Female	55	No education	Yes

Four themes emerged from the data: patients' views about unused medications; reasons for ending up with unused medications; storage and disposal practices; and potential solutions for medication waste minimisation (Table 2).

**Table 2:** Summary of themes, sub-themes, and categories identified in the analysis.

Theme	Sub-theme	Categories
Patients' views on unused medications	Positive views	Unused medications are useful for future disease management
		Avoiding visits to a distant health facility
	Negative views	Endangering health
		Polluting the environment
		Financial loss
Reasons for ending up with unused medication	Non-adherence	Side effects
		Feeling better
		Perceived ineffectiveness
		Severity of the disease
		Lack of knowledge
	Health-system related factors	Lack of trust in healthcare services
		Distance from home to a health facility
		Easy access
		Excess supply
	Patient-healthcare provider relationships	Lack of trust in healthcare providers' competence
		Fearing healthcare providers
		Not accounting for patient preferences
Storage and disposal of unused medications	Storage	Storage location
		Storage duration
		Commonly stored unused medication
	Disposal	Disposal method
		Reason for disposal method
Potential solutions	Education and counselling	Education on appropriate use of medications
		Counselling on the benefits of finishing prescribed medications and the risks of early discontinuation
		Taking responsibility
	System strengthening	Diagnostic capacity of health facilities
		Improving providers' competence
		Packaging
		Quality of care

### 3.1 Patients' views on unused medications

Patients had mixed views about unused medications. While most patients disagreed with keeping unused medications at home, others were positive or indifferent—often entirely unaware of the potential consequences of doing so.

#### 3.1.1 Positive views

Patients with positive perceptions stated that keeping unused medication at home could be useful for managing future diseases. They believed that if a medication had previously provided a positive therapeutic effect, it could offer immediate relief during acute episodes, such as severe pain, or for recurrent conditions. Patients also felt that keeping medication at home could save them from travelling to a distant healthcare facility to obtain a new prescription.

*I could reuse the leftovers if I have similar symptoms. Since the medicine worked the last time I took it, it will save me another trip to a health facility* (Participant 1)*.*

*I have asked my sister to share her medication for headaches with me. As she frequently comes to the health centre for treatment, she has leftover medications that she keeps. I took what she gave me. By the time I fell ill, it was already dark, and we live far from the health centre (Participant 5).*

#### 3.1.2 Negative views

Patients with negative views about unused medications expressed concerns over potential health risks to humans and animals. They noted that vulnerable groups —such as children, the elderly, individuals with limited literacy, and family pets, could be accidentally poisoned from leftover medications.

*My husband was given a cough syrup, but after taking it once or twice, his vision became blurred, and he experienced mental instability. Suspecting that the medication might be incorrect, we told him to stop taking it. While playing, my child found the medication and drank it. I did not see it happen, but his friends did. He became extremely sick. I gave him ash, egg, and milk, and that is how he recovered. Taking medication meant for another person is not good* (Participant 14)*.*

Furthermore, patients anticipated the risk of unused medications being used outside their intended purpose, endangering both themselves and others. They noted that the presence of unused medication could tempt other family members to self-medicate without a proper diagnosis. Patients also recognised the risk for environmental pollution if these medications were improperly disposed of.

*I have observed the misuse of medications by people around me. For example, they apply antibiotic capsule powder directly onto wounds. They also make chickens swallow these medications* (Participant 19)*.*

*Once, my daughter was sick, and she took the medication I had brought home from the health centre. She thought it would be all right to do that. What if it killed her or made her sick? I told her she should never do this and explained that what is prescribed for me might not be okay for her because we are not the same age* (Participant 3)*.*

Some patients regarded obtaining medication from a health facility and then leaving it unused as an economic loss.

*I am very much concerned when I see leftover medications, even when I see people keeping medications inappropriately. I tell them, “How do you expect to get cured by the medicine if you store it this way? Why do you take government medicine only to keep it at home? Why waste the money?* (Participant 7)*.*

### 3.2 Reasons for ending up with unused medication

Medications remained unused in households for various reasons, including non-adherence, inadequate patient-healthcare provider relationships, and factors related to the health system.

#### 3.2.1 Non-adherence

Most patients reported not taking medications as prescribed. Reasons for non-adherence behaviour were intentional, such as experiencing side effects, feeling better, perceived treatment ineffectiveness, perceived severity of the illness (e.g., thinking a disease is minor), and a lack of knowledge.

*Well, for me, the point of going to the hospital is to get better. I want to feel better and get back to work as soon as possible. However, some medications cause gastric irritation. Furthermore, they do not always settle peacefully within our bodies. In such cases, I stop taking the medication* (Participant 2)*.*

*Most of the time, when I have a serious illness, I finish my medication exactly as prescribed. But if the condition is minor, I might not finish the medication* (Participant 25)*.*

*I just do not like taking pills in general; I prefer to be prescribed an injection, but that never happens. When it comes to injections, I will have already taken the dose while at the clinic, and it will certainly work. However, with the pills, I know I will take them home and skip a few doses, so I am not optimistic about them* (Participant 1)*.*

Most patients received limited and sometimes conflicting information from various sources, including healthcare providers, families, and friends about their diagnosis and prescribed medication, particularly on the importance of completing the full course and safely disposing of medication. This conflicting information confused patients and led some to discontinue their medication.

*I took my wife to the health center because she was experiencing swelling, itching, and a burning sensation in her uterus. When I spoke to the health professional, he told me that it was a STD. I asked what the origin of the disease because the professional had only given me a written note and a referral. No detailed explanation of what the disease was had been given to me. We were then sent to the pharmacy, where we were told to take the medication ‘one in the morning, one in the afternoon, and one at night.’ But nothing was clear. She was given an injection and a medication. She got worse. I told them that I wanted a proper response. I think health centers need attention* (Participant 23)*.*

#### 3.2.2 Health-system related factors

Health system-related factors such as a lack of trust in healthcare services, long distance to health facilities, and excess supply of medications were cited as reasons for ending up with unused medications.

Patients perceived that public health facilities in rural areas were ill-equipped with medical devices and other inputs necessary for diagnosing, monitoring, and treating illness. They also felt that medications provided under the community-based insurance system were of lower quality. This lack of trust in the quality of public healthcare services ultimately led some patients to end up with unused medications.

*The doctors do not have all the medical equipment necessary for diagnosis. They do not have working X-ray machines or all the equipment they need to make a complete diagnosis. So, some patients, even if they collect the medication, may not take it as prescribed because they feel the doctors do not treat them properly* (Participant 3).

The distance from home to the nearest public health facility was also cited as a reason why some patients purchased and stockpiled excess medications, leading to waste.

*If we start our journey by 2 p.m., we will arrive at the public health facility by 5 p.m. It is uphill and difficult. Sometimes, it is already too late to collect the medicine by the time we arrive, which is quite a challenge. So, I sometimes buy medicine for stomach aches, cramps, or headaches from private pharmacies on the advice of a physician* (Participant 18).

Some patients visited health facilities frequently because they were members of a community-based health insurance scheme, which gave them easy access to medications and made wasteful behaviour likely.

*To be honest, the issue of how much money is spent on medicine never crosses our minds. We simply perceive it as free medication since the government provides it free of charge. I take the medicine I've paid for out of pocket more strictly* (Participant 1).

*Ever since this ‘Tena Medihin (community-based health insurance)’ program started, I hear people saying, ‘I have already paid for it once,’ so they do not really consider it as their own loss* (Participant 19).

A few patients perceived that their medications remained unused because healthcare providers had supplied more medication than was needed.

*Even if it was after nine months of gestation and some time was left before due date; they gave me three or four packs of amoxicillin. That is too much* (Participant 1).

#### 3.2.3 Patient-healthcare provider relationships

Patient-healthcare provider relationships, such as trust in the provider's competence, communication, fear, decision-making, and consultation length, were found to be linked with unused medications. Patients, particularly those from remote areas, expressed lower trust in younger healthcare providers, whom they perceived as inexperienced and less knowledgeable.

*People say that all the healthcare professionals these days are just kids, that there are not even any adults, and that they are uncomfortable with that* (Participant 3)*.*

Patients who felt that they had waited long hours and received less care, time, and respect during patient-provider encounters reported keeping their medication unused.

*What discourages me from coming to the clinic is that I fear that the people who work there might think that I come too often. Let us say that I visited the clinic and a week later I got sick again, but I will not go for a check-up because I do not feel comfortable going. Also, there is a clear difference between the medical treatment we receive through health insurance versus paying out of pocket. When we are sent for treatment through health insurance referrals, the process can take two days or more; you might not be lucky. By contrast, if you go to a private clinic, you will see a doctor in seconds, take your medicine, and head back home with nice treatment and care* (Participant 2)*.*

### 3.3 Storage and disposal of unused medications

#### 3.3.1 Storage

Patients frequently reported storing unused medication at home for a period of up to three months. Home storage depended on the type of medication; for instance, painkillers were commonly retained for potential future use. Within the household, patients kept unused medication in the living room, bedroom, or kitchen. These medications were kept in variety of places, including a closed storage box, luggage, drawers, a traditional food container “*Agelgil*,” kitchen cupboards, under the mattress, and in a refrigerator.

*I keep medication for a month. If it stays longer than a month, I come and collect a new one because I think its strength will have decreased* (Participant 2).

#### 3.3.2 Disposal

Most patients reported disposing of unused medications by throwing them into an open field, keeping them in a household dustbin, burning them with trash, flushing them down toilets, or dumping them in ditches. Little or no attention was paid to environmental contamination when doing so. However, some patients reported mixing unused medications with household rubbish or animal dung during disposal to prevent them from being accidentally swallowed by vulnerable groups such as children and cattle.

*I think that discarding them (unused medication) in the toilet does not pollute. If I discard them in the toilet, how can it (pollute)?* (Participant 24).

One patient disposed of unused medications at a church, believing that her illness had been caused by an evil spirit and could not be treated with modern medications.

### 3.4 Potential solutions

Patients proposed various solutions to prevent or minimize medication waste at home. These solutions targeted patients themselves, the patient-provider relationship, and healthcare system.

#### 3.4.1 Education and counselling

Educating patients about the appropriate use of medications was proposed as a vital solution for achieving better therapeutic outcomes and reducing wastage. Patients suggested delivering this education in a range of settings. These included health facilities while patients were waiting to see a doctor, and at *Edir* gatherings (i.e., a traditional mutual aid organization). Leveraging other community gathering places and implementing house-to-house visits by health extension workers were highlighted as means of health education.

*I advise patients to listen to healthcare professionals' advice. For example, if they tell me to take two (pills) now, then two in the evening, two in the middle of the night, and two in the morning, for five days, I will take them all and finish the course. If I do not get better soon, then I will go back and tell them that I have been really sick and ask them for either different pills or an injection* (Participant 3).

*It would be better if they (doctors) took this opportunity (*i.e.*, the high patient load and long waiting time) to educate or train us while we're gathered. They could pre-select two or three doctors and educate us on proper medication use* (Participant 22)*.*

During clinical counselling, patients expressed a need for providers to address their questions and concerns, follow-up on their therapeutic progress, and advise them on the benefits of completing prescribed medication therapy. In addition, most patients suggested that written medication instructions prepared in the local language should be given. Most patients advised others to act responsibly to reduce or prevent medication waste. They said patients should follow healthcare providers' advice, take medication as prescribed, and refuse to collect medication if they are not going to take it.

*Why do patients not refuse to take their prescribed medication instead of receiving it if they know they will not use it and will throw it away, so that at least someone else can make use of it and get better?* (Participant 3).

#### 3.4.2 System strengthening

Equipping public health facilities with the necessary diagnostic equipment was proposed as a solution that would avoid the need for providers to prescribe medications on the basis of symptoms and history alone. This, in turn, would help patients to trust the diagnosis and to take the prescribed medications as directed.

*I am sure that if we really looked into the health centre, we would find a lot of equipment and devices lacking. But they run laboratory tests for us. For example, they don't have suture threads, and they don't have an X-ray machine. An X-ray machine would be extremely useful for this health centre, but they don't have one, and there is much else they lack* (Participant 2)*.*

*You know that feeling of satisfaction when you see your X-ray result and go, ah, that is my disease. It is nice to know exactly what disease you have and to get your blood pressure measured* (Participant 13).

Improving providers' competence, particularly their technical, communication, care, and service-provision skills, was identified as an intervention to reduce medication waste. Additionally, a few patients suggested optimizing packing, issuing paper bags, limiting the quantity of the medication dispensed, and allowing unused medications in good condition to be returned so that they could be made available to others, thereby preventing waste.

## 4 Discussion

Patients' views on unused medications were explored in this study, where some had favourable perceptions and others had negative views towards unused medications kept at home. Patients with favourable perceptions are more likely to keep unused medications for future use.^36^ This has been related to patients' income levels,^28^ education levels, awareness of the medications they take,^37^ and proper storage practice at home.^38^ Non-adherence to prescribed medication regimens due to side effects, feeling better, perceived ineffectiveness, and lack of knowledge leads to medications ending up unused, which is congruent with previous studies.^5^, ^37^, ^39^, ^40^, ^41^, ^42^

Most patients lack knowledge about their diagnosis, the name and indication of the prescribed medication, the risks of not finishing a course of medication, and how to dispose of it safely. Studies show that patients are not adequately counselled,^43^, ^44^ particularly on the benefits of completing prescribed medication regimens and on what to do if a medication remains unused at home.^45^

In addition, some patients develop perceptions that hinder them from taking their medication as prescribed. These include, judging the competence of healthcare providers by their age, experience, and communication style; perceiving that excess medication is supplied^38^, ^40^, ^41^ and expecting that diagnostic tests and procedures should be performed for all illnesses, including minor ones, before medication is initiated. Where treatment is initiated empirically without such diagnostic confirmation, patients are less inclined to take their medication as prescribed, which contributes to early discontinuation and, in turn, to unused medications.

Providers also need to consider the patient's adherence, the cost of medication, and the possibility that unused medication is already present at the patient's home before deciding the quantity to be supplied. They should also encourage patients to finish their medication, to return unwanted medicines to their community pharmacy where a take-back program exists, or otherwise to dispose of medication safely.^10^ In addition, healthcare providers must be mindful of their relationship with patients while providing care. By treating all patients equally and with respect, and listening to their concerns, they can strengthen patients' trust, which is positively associated with improved health^46^, ^47^, ^48^ and behavioural outcomes,^49^ such as treatment adherence,^9^ and reduced wastage.^50^ As patients' preferred source of information, providers need to keep their knowledge up to date as well as control their self-interest to avoid mistrust and maximise treatment outcomes including reducing unused medication.^51^

Moreover, interventions aimed at enhancing health facility governance—specifically by establishing transparent communication, demonstrating competence, and ensuring service quality—are critical to gaining patient trust.^52^ Creating a favourable working environment that enhances healthcare providers' job satisfaction not only improves their professional performance but also increases patient satisfaction. This study documented the perceived health, environmental, and economic risks of unused medication on communities. Most medications were disposed of by being thrown into open fields. However, some good practices were also observed. This includes mixing unused medications with household trash or animal dung during disposal to avoid accidental swallowing of unused medications by vulnerable groups such as children and cattle, which is in line with recommended safe disposal practice.^53^

Minimizing unused medications and their associated risks requires multiple interventions.^21^ These include education, persuasion, incentivisation, coercion, training, restrictions, environmental restructuring, modelling and enablement. Implementing these interventions effectively may also need guidelines, environmental or social planning, communication, legislation, service provision, regulation, and fiscal measures.^54^ Furthermore, these interventions can target manufacturers, distributors, prescribers, dispensers, patients, and health authorities.^21^

In resource constrained countries like Ethiopia, evaluating the appropriateness of existing or proposed interventions is critical.^55^ Key priorities include improving stock management, ensuring rational prescribing and dispensing practices, optimizing dispensing processes, and raising patients' awareness. For example, using technology such as a medication-sharing app was found to help reduce wastage by streamlining medication counselling, sharing, and recycling among community members, pharmacies, and government agencies.^56^ At present, however, there is no drug take-back program in Ethiopia, nor are there interventions aimed at reducing unused medications among patients. This study identifies modifiable gaps in health education and literacy, in the patient-provider relationship (i.e., the social environment), and in psychological factors, around which interventions can be designed and implemented. Few disposal interventions have been reported in the literature from low- and middle-income countries,^57^, ^58^, ^59^, ^60^ and those that have been reported highlight intensified counselling and education, the promotion of rational prescribing and dispensing, the optimisation of dispensing processes and the improvement of patient-provider relations as ways to prevent or minimize medication waste.

### 4.1 Strengths and limitations

This study has some limitations that should be considered when interpreting its results. First, because of its qualitative methodology, limited geographic coverage (due to the conflict in various parts of the country), and focus on primary health care units only, the results may not be well generalized to other settings. However, findings provide patients' views that are not well-described in the existing literature base.

On the other hand, the study's strength lies in its inclusion of rural patients' perspectives, who account for 70% of the total population in Ethiopia. In addition, these patients were recruited from health centres, which are the primary point of contact for most patients to get basic healthcare services.

**Conclusion:** Patients' perceptions of unused medications kept in households are mixed. While some view leftover medications as valuable for managing future illness, others emphasise the dangers that keeping medications poses to humans, animals, the environment, and the economy. Further studies focusing on providers are needed to gain a comprehensive perspective. Reducing unused medications in households will require interventions that target patients' medication use behaviour, providers' prescribing and dispensing practices, and the overall quality of care delivered by the health system. Such interventions should be designed to strengthen patients' trust in the quality of care they receive, since that trust in turn shapes how patients use their medication and whether they dispose of it safely and responsibly.

## CRediT authorship contribution statement

**Dawit T. Gebregeorgise:** Writing – original draft, Validation, Methodology, Investigation, Funding acquisition, Formal analysis, Data curation, Conceptualization. **Teferi G. Fenta:** Writing – review & editing, Validation, Supervision, Methodology, Investigation, Formal analysis, Data curation, Conceptualization. **Bart J.F. van den Bemt:** Writing – review & editing, Validation, Supervision, Resources, Project administration, Methodology, Investigation, Formal analysis, Data curation, Conceptualization. **Charlotte L. Bekker:** Writing – review & editing, Validation, Supervision, Resources, Project administration, Methodology, Investigation, Formal analysis, Data curation, Conceptualization.

## Consent for publication

Not applicable.

## Ethics approval and consent to participate

The Ethics Committee of the School of Pharmacy, Addis Ababa University, approved this study [ERB/SOP/466/15/2023]. The North Shoa Zonal Health Bureau, the Woreda health bureaus, and the heads of the selected health centres granted permission for the study to be conducted. After purpose of the study and the anonymity of reporting had been explained, verbal informed consent was obtained from all volunteering patients before participation.

## Declaration of generative AI and AI-assisted technologies in the writing process

During the preparation of this work, the author used ChatGPT-4.5 and Claude (Anthropic) to improve language and readability of the manuscript. After using these tools, the authors reviewed and edited the content as needed and take full responsibility for the content of the publication.

## Funding

This research was supported by an unrestricted grant from LEARN Logistics by the Kühne Foundation through the Senselet project.

## Declaration of competing interest

The authors declare no competing interests.

## Data Availability

No datasets were generated or analyzed during the current study.
